# α-MSH and Foxc2 promote fatty acid oxidation through C/EBPβ negative transcription in mice adipose tissue

**DOI:** 10.1038/srep36661

**Published:** 2016-11-07

**Authors:** Lu Gan, Zhenjiang Liu, Yizhe Chen, Dan Luo, Fei Feng, Guannv Liu, Chao Sun

**Affiliations:** 1 College of Animal Science and Technology, Northwest A&F University, Yangling, Shaanxi, 712100, China

## Abstract

Alpha melanocyte stimulating hormone (α-MSH) and Forkhead box C2 protein (Foxc2) enhance lipolysis in multiple tissues. However, their relationship in adipose fatty acid oxidation (FAO) remains unclear. Here, we demonstrated that α-MSH and Foxc2 increased palmitate oxidation to CO_2_ in white (WAT) and brown adipose tissue (BAT). C/EBPβ expression was reduced by α-MSH and Foxc2. FFA level was elevated by α-MSH and pc-Foxc2 treatment along with increased FAO in white and brown adipocytes. The expression of FAO key enzymes, medium-chain acyl-CoA dehydrogenase (MCAD) and long-chain acyl-CoA dehydrogenase (LCAD) were increased in α-MSH and pc-Foxc2 group. Combination of α-MSH and Foxc2 treatment synergistically promoted FAO through increasing the activity of CPT-1 and phosphorylation of ACC. We found C/EBPβ bind to MC5R and Foxc2 promoter regions and inhibited FAO. cAMP level was increased by α-MSH and Foxc2 individually treated or combined treatment. Furthermore, cAMP/PKA pathway-specific inhibitor (H89) blocked the FAO, despite in α-MSH and Foxc2 both added group. While forskolin, the cAMP agonist, promoted FAO and enhanced the effect of α-MSH and Foxc2. Collectively, α-MSH and Foxc2 mutual promote FAO in WAT and BAT via cAMP/PKA signal pathway. And C/EBPβ as a transcription suppressor inhibits α-MSH and Foxc2 expression and FAO.

Obesity, type 2 diabetes, cardiovascular and cerebrovascular diseases are increased risks for human health. Promotion of cellular lipid metabolism comes to be a valid therapeutic strategy to treat these diseases^1^. Adipose tissue with large amount of triglycerides is a target in regulating fat deposition and energy expenditure. Melanocortin system plays a pivotal role in controlling food intake, body weight and energy homeostasis^2^,^3^,^4^. In central nervous system (CNS), the melanocortin-3 and -4 receptors (MC3R/MC4R) have been demonstrated to regulate body weight, energy homeostasis, and feeding behavior^5^,^6^. Mice and humans lacking MC4R lead to significant obesity and dysregulated energy balance^7^,^8^.

Melanocortins also affects energy homeostasis by acting on peripheral tissues. Alpha melanocyte stimulating hormone (α-MSH), produced by proopiomelanocortin (POMC) neurons, act via the interaction with its receptors in reducing food intake and increasing energy consumption of the peripheral tissues and organs^9^,^10^,^11^. Studies show that the peripheral administration of α-MSH analogue effectively reduced weight in diet-induced obese mice^12^. Malonyl-CoA is an inhibitor of carnitine palmitoyltransferase-1 (CPT-1), controls the transfer of long-chain fatty acyl-CoA molecules into mitochondria for oxidation^13^. And fatty acid oxidation (FAO) is partly regulated by malonyl-CoA, which is synthesized from cytosolic acetyl-CoA via a catalyzed reaction by acetyl-CoA carboxylase (ACC). Leptin stimulates FAO and glucose uptake, and prevents the accumulation of lipids via activation of PKA and the inhibition of ACC^14^. POMC gene expression is reduced in both db/db and ob/ob mice, and treatment with leptin increases the mRNA expression of POMC, which shows a positive connection between leptin and α-MSH^15^. In murine 3T3-L1 adipocytes, melanocortins are known to activate the PKA and ERK1/2 signaling pathways and stimulate the activity of ATGL which directly regulates lipolysis^16^,^17^,^18^,^19^. Rodrigues *et al*.^20^ indicate that α-MSH signaling promotes lipolysis and impairs re-esterification in 3T3-L1 adipocytes via melanocortin 5 receptor (MC5R). However, whether α-MSH stimulates fatty acid oxidation in adipose tissue remains unclear, even though α-MSH has been shown to have direct effects on lipid metabolism^20^,^21^,^22^.

Forkhead box C2 protein (Foxc2) is a member of the forkhead/winged helix transcription factor family in various organs and tissues, such as cardiac system, mammary gland, liver and adipose tissue. Our previous studies found Foxc2 enhanced mice pre-adipocytes proliferation and inhibited apoptosis via activating Akt/mTORC1 signaling pathway^23^. Foxc2 stimulates osteogenic differentiation and inhibits adipogenic differentiation in bone marrow mesenchymal stem cells, and in white adipocytes^24^,^25^. Overexpression of Foxc2 in adipocytes prevents diet-induced obesity and insulin resistance^26^,^27^. Interestingly, in our research we found Foxc2 reduces the formation of lipid droplets and inhibits differentiation in both 3T3-L1 adipocytes and primary adipocytes. But the regulatory role of Foxc2 on adipose tissue fatty acid oxidation remains elusive.

Here, we provide evidences for the profound effects of C/EBPβ in α-MSH-mediated and Foxc2-mediated fatty acid oxidation in both white and brown adipose tissues. Furthermore, we show that cAMP/PKA signal pathway which activated by α-MSH and Foxc2 is essential in stimulating FAO in adipose tissues.

## Results

### α-MSH promotes fatty acid oxidation in WAT and BAT

**Figure 1:**
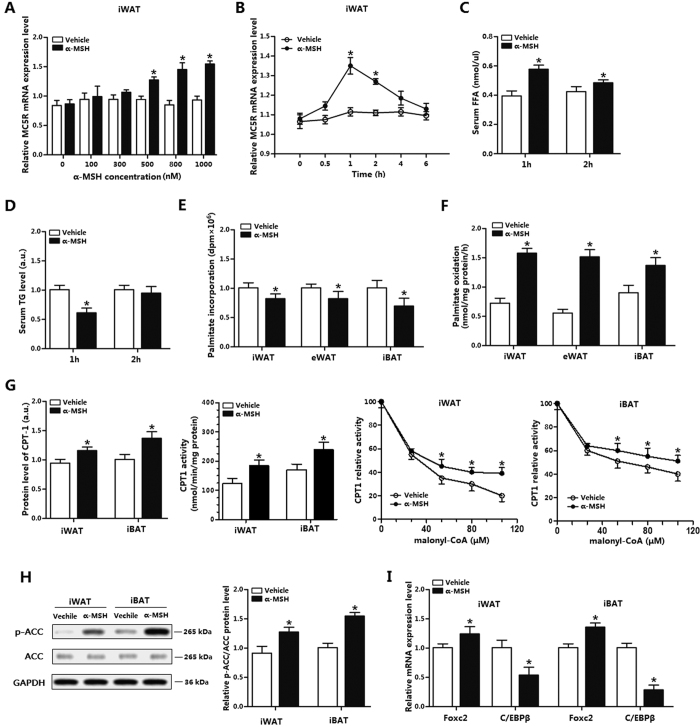
α-MSH promotes fatty acid oxidation in WAT and BAT. (**A**) MC5R mRNA in iWAT after 0 nM, 100 nM, 300 nM, 500 nM, 800 nM and 1000 nM α-MSH injection for 1 h (n = 6); (**B**) MC5R mRNA in iWAT at 0 h, 0.5 h, 1 h, 2 h, 4 h and 6 h after 500 nM α-MSH injection (n = 6); (**C**) Serum FFA level of mice after 500 nM α-MSH injection for 1 h and 2 h (n = 10); (**D**) Serum TG level of mice after 500 nM α-MSH injection for 1 h and 2 h (n = 10); (**E**) Palmitate incorporation into different locations of adipose tissues after 500 nM α-MSH injection for 1 h (n = 6); (**F**) Relative palmitate oxidation to CO_2_ (n = 6); (**G**) Effects of α-MSH on CPT-1 in iWAT and iBAT. 500 nM α-MSH injection into mice for 1 h and CPT-1 level was detected by ELISA; CPT-1 activity and sensitivity were detected by the method mentioned in METHODS (n = 6); (**H**) Effects of α-MSH on phosphorylation of ACC in iWAT and iBAT (the full-length blots of ACC and p-ACC were showed in Figure S6A). 500 nM α-MSH injection into mice for 1 h (n = 6); (**I**) mRNA of Foxc2 and C/EBPβ in iWAT and iBAT. 500 nM α-MSH injection into mice for 1h (n = 6). CPT-1: carnitine palmitoyl transferase-1, ACC: acetyl-CoA carboxylase, Values are means ± SD. vs. control group, *p *<* 0.05.

To study the effects of α-MSH on fatty acid oxidation in mice adipose tissue, we intraperitoneally injected α-MSH into male mice. Figure 1A,B indicated 500 nM of α-MSH injection for 1 h increased MC5R. Serum α-MSH level was increased after injection (Figure S1). α-MSH injection for 1 h elevated the release of serum FFA, while reduced the level of TG (Fig. 1C,D). And α-MSH injection for 2 h still increased serum FFA level but did not alter serum TG level (Fig. 1C,D). The assay about the incorporation of [^14^C]-palmitate into different adipose tissues (iWAT, eWAT and iBAT) showed α-MSH injection remarkably produced a 0.3-fold to 0.5-fold decrease in the palmitate incorporation into iWAT, eWAT and iBAT (Fig. 1E). In addition, the production of [^14^C]-palmitate oxidation into CO_2_ was higher in iBAT than that in WAT (Fig. 1F). CPT-1 was elevated in iWAT and iBAT after α-MSH injection (Fig. 1G). CPT-1 activity and CPT-1 sensitivity to malonyl-CoA inhibition were higher in α-MSH injected group compared with controls (Fig. 1G). Consistently, phosphorylation level of ACC was increased as shown in Fig. 1H. Foxc2 increased in adipose tissue by α-MSH treatment. Conversely, CEBP/β level was reduced in iWAT and iBAT (Fig. 1I). Thus, α-MSH exhibits positive regulation on fatty acid oxidation and implies a relationship between Foxc2 and CEBP/β.

### Foxc2 increases fatty acid oxidation in WAT and BAT

**Figure 2:**
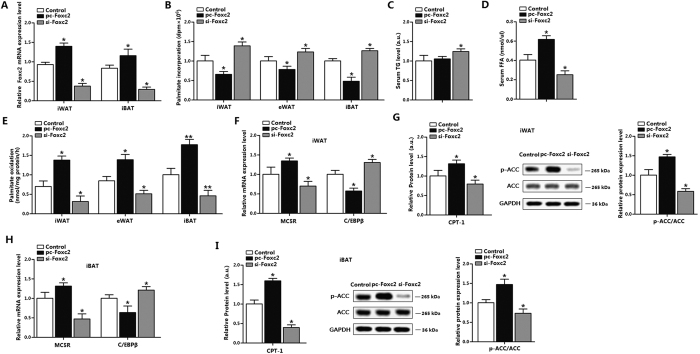
Foxc2 increases fatty acid oxidation in WAT and BAT. (**A**) Foxc2 level in iWAT and iBAT after pc-Foxc2 or si-Foxc2 injection for 6 days (n = 6); (**B**) Palmitate incorporation into different locations of adipose tissues after pc-Foxc2 or si-Foxc2 injection for 6 days (n = 6); (**C**) Serum TG level of mice after pc-Foxc2 or si-Foxc2 injection for 6 days (n = 10); (**D**) Serum FFA level of mice after pc-Foxc2 or si-Foxc2 injection for 6 days (n = 10); (**E**) Relative palmitate oxidation to CO_2_ in different adipose tissues (n = 6); (**F**) MC5R and C/EBPβ levels of iWAT after pc-Foxc2 or si-Foxc2 injection for 6 days (n = 6); (**G**) Protein levels of CPT-1 and phosphorylation level of ACC (the full-length blots of ACC and p-ACC were showed in Figure S6B) in iWAT after pc-Foxc2 or si-Foxc2 injection for 6 days (n = 6); (**H**) MC5R and C/EBPβ levels of iBAT after pc-Foxc2 or si-Foxc2 injection for 6 days (n = 6); (**I**) Protein levels of CPT-1 and phosphorylation level of ACC (the full-length blots of ACC and p-ACC were showed in Figure S6B) in iBAT after pc-Foxc2 or si-Foxc2 injection for 6 days (n = 6). pc-Foxc2: adenovirus overexpression vector of Foxc2, si-Foxc2: lentiviral interference vector of Foxc2, CPT-1: carnitine palmitoyl transferase-1, ACC: acetyl-CoA carboxylase, the protein expression was detected by ELISA test. Values are means ± SD. vs. control group, *p *<* 0.05, **p *<* 0.01.

To clarify the function of Foxc2 on fatty acid oxidation, we used the exogenous gene expression method by intraperitoneal injection of pc-Foxc2 or si-Foxc2. Data showed Foxc2 increased effectively in iWAT, iBAT and liver (Fig. 2A and S2A). The incorporation of [^14^C]-palmitate detection and fatty acid oxidation measurement showed Foxc2 promoted fatty acid oxidation in iBAT more effectively than that in WAT (Fig. 2B,E). Serum TG content increased slightly in pc-Foxc2 group compared with that in control group (Fig. 2C) and FFA level was increased remarkably (Fig. 2D). Foxc2 elevated the MC5R level, but not MC3R and MC4R in iWAT (Figure S2B); and down-regulated CEBP/β in both iWAT and iBAT, suggesting a negative correlation between Foxc2 and MC5R (Fig. 2F,H). The injection of pc-Foxc2 up-regulated the protein level of CPT-1 and increased the phosphorylation level of ACC in both iWAT and iBAT (Fig. 2G,I). And also CPT-1 activity was increased in pc-Foxc2 group, and decreased in si-Foxc2 group of iWAT and iBAT; CPT-1 sensitivity measurement showed the same result (Figure S2C,D). Moreover, the function of Foxc2 on fatty acid oxidation was more obvious in iBAT, and this is consistent with the function of α-MSH in iBAT. Taken together, these discoveries suggest Foxc2 has active impacts on adipose fatty acid oxidation.

### α-MSH and Foxc2 jointly increase fatty acid oxidation in white and brown adipocytes

**Figure 3:**
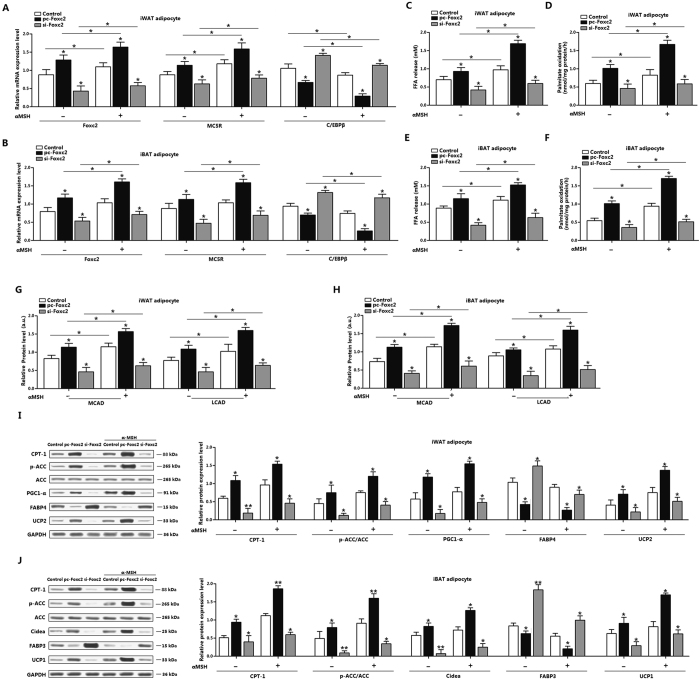
α-MSH and Foxc2 jointly increase fatty acid oxidation in white and brown adipocytes. (**A**) mRNA levels of Foxc2, MC5R and C/EBPβ of iWAT adipocytes. adipocytes were pre-transfected with pc-Foxc2 and si-Foxc2 for 72 h and and then treated with 500 nM α-MSH for 1h before collected (n = 3); (**B**) mRNA levels of Foxc2, MC5R and C/EBPβ of iBAT adipocytes. Adipocytes were pre-transfected with pc-Foxc2 and si-Foxc2 for 72 h and then treated with 500 nM α-MSH for 1h before collected (n = 3); (**C**) FFA level of iWAT adipocytes with pc-Foxc2 or si-Foxc2 transfection for 72 h and 500 nM α-MSH incubation for 1 h (n = 3); (**D**) Palmitate oxidation of iWAT with pc-Foxc2 or si-Foxc2 transfection for 72 h and 500 nM α-MSH incubation for 1 h (n = 3); (**E**) FFA level of iBAT adipocytes with pc-Foxc2 or si-Foxc2 transfection for 72 h and 500 nM α-MSH incubation for 1 h (n = 3); (**F**) Palmitate oxidation of iBAT with pc-Foxc2 or si-Foxc2 transfection for 72 h and 500 nM α-MSH incubation for 1 h (n = 3); (**G**) Levels of MCAD and LCAD of iWAT adipocytes with pc-Foxc2 or si-Foxc2 transfection for 72 h and 500 nM α-MSH incubation for 1 h, protein level was detected by ELISA test (n = 3); (**H**) Levels of MCAD and LCAD of iBAT adipocytes with pc-Foxc2 or si-Foxc2 transfection for 72 h and 500 nM α-MSH incubation for 1 h, protein level was detected by ELISA test (n = 3); (**I**) Protein levels of CPT-1, p-ACC, PGC1-α, FABP4 and UCP2 in iWAT with pc-Foxc2 or si-Foxc2 transfection for 72 h and 500 nM α-MSH incubation for 1 h (n = 3); (**J**) Protein levels of CPT-1, p-ACC, Cidea, FABP3 and UCP1 in iWAT with pc-Foxc2 or si-Foxc2 transfection for 72 h and 500 nM α-MSH incubation for 1 h (n = 3). pc-Foxc2: adenovirus overexpression vector of Foxc2, si-Foxc2: lentiviral interference vector of Foxc2, CPT-1: carnitine palmitoyl transferase-1, ACC: acetyl-CoA carboxylase, ACC: acetyl-CoA carboxylase. Values are means ± SD. vs. control group, *p *<* 0.05.

We then examined the relationship between Foxc2 and α-MSH during fatty acid oxidation in adipocytes. Foxc2 elevated MC5R level, and the addition of α-MSH further increased the level of MC5R in iWAT and iBAT adipocytes; and reduced CEBP/β more effectively than Foxc2 alone (Fig. 3A,B). In addition, Foxc2 and α-MSH co-treatment elevated the release of FFA, and [^14^C]-palmitate oxidation to CO_2_ in both iWAT and iBAT adipocytes (Fig. 3C–F), which were consistent with the *in vivo* experiment. The MCAD and LCAD levels of iWAT and iBAT adipocytes were both increased in the co-treatment group (Fig. 3G,H). Then we detected the key fatty acid oxidation proteins in iBAT and iWAT adipocytes, Foxc2 elevated the levels of CPT-1, p-ACC, PGC1-α and UCP2, but reduced FABP4 level in iWAT adipocytes (Fig. 3I). And interfered of Foxc2 increased the level of FABP4, but reduced the other proteins (Fig. 3I). The addition of α-MSH reinforced the increasing of CPT-1, p-ACC, PGC1-α and UCP2, suggesting Foxc2 and α-MSH promote fatty acid oxidation synergistically. Consistently with the results of iWAT, Foxc2 elevated CPT-1, p-ACC, Cidea and UCP1 with the incubation of α-MSH in iBAT adipocytes, while accentuated the reduction of FABP3 (Fig. 3J). We then measured the CPT-1 activity in adipocytes. Results showed CPT-1 activity was increased with pc-Foxc2 treatment in the two kind of adipocytes; and the addition of α-MSH elevated the activity of CPT-1 (Figure S3A,C). And the CPT-1 became more sensitivity to malonyl-CoA inhibition after α-MSH addition in both iWAT and iBAT adipocytes which were pre-treated with pc-Foxc2 (Figure S3B,D). Hence we conclude Foxc2 and α-MSH promote fatty acid oxidation cooperatively in two kinds of adipocytes.

### C/EBPβ reduces adipocytes fatty acid oxidation by negative transcriptional regulation of MC5R

**Figure 4:**
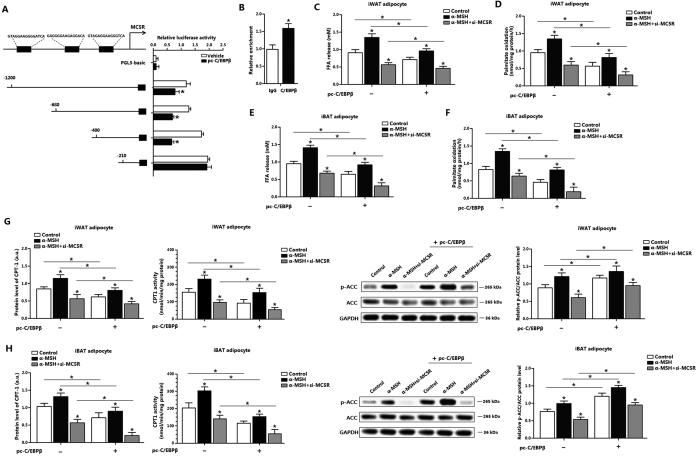
C/EBPβ reduces adipocytes fatty acid oxidation by negative transcriptional regulation of MC5R. (**A**) Fragments of MC5R promoter fused to a luciferase reporter gene were co-transfected into HEK293T cells together with PGL3-basic (control) or pc-C/EBPβ (n = 3). Luciferase activity was corrected for Renilla luciferase activity and normalized to control activity (n = 3). (**B**) Chromatin immunoprecipitation (ChIP) analysis of MC5R and C/EBPβ (n = 3). (**C**) Effects of C/EBPβ on FFA level in iWAT adipocytes. Cells were pre-transfected with pc-C/EBPβ or si-MC5R, and then incubated with 500 nM α-MSH for 1 h (n = 3); (**D**) Effects of C/EBPβ on palmitate oxidation in iWAT adipocytes. Cells were pre-transfected with pc-C/EBPβ or si-MC5R, and then incubated with 500 nM α-MSH for 1 h (n = 3); (**E**) Effects of C/EBPβ on FFA level in iBAT adipocytes. Cells were pre-transfected with pc-C/EBPβ or si-MC5R, and then incubated with 500 nM α-MSH for 1 h (n = 3); (**F**) Effects of C/EBPβ on palmitate oxidation in iBAT adipocytes. Cells were pre-transfected with pc-C/EBPβ or si-MC5R, and then incubated with 500 nM α-MSH for 1 h (n = 3); (**G**) Effects of C/EBPβ on the protein expression of CPT-1, CPT-1 activity and phosphorylation level of ACC in iWAT adipocytes. Cells were pre-transfected with pc-C/EBPβ or si-MC5R, and then incubated with 500 nM α-MSH for 1 h (n = 3); (**H**) Effects of C/EBPβ on the protein of CPT-1, CPT-1 activity and phosphorylation level of ACC in iBAT adipocytes. Cells were pre-transfected with pc-C/EBPβ or si-MC5R, and then incubated with 500 nM α-MSH for 1 h (n = 3). pc-C/EBPβ: pcDNA3.1-C/EBPβ overexpression vector of C/EBPβ, si-MC5R: lentiviral interference vector of MC5R, CPT-1: carnitine palmitoyl transferase-1, ACC: acetyl-CoA carboxylase, the protein level of CPT-1 was detected by ELISA test. Values are means ± SD. vs. control group, *p *<* 0.05.

We next explored whether C/EBPβ interacted with MC5R physically. With Genomatix software analysis, we found three potential binding sites of C/EBPβ on the MC5R promoter region (Fig. 4A). Then by the luciferase reporter assay, the −400~−210 region was considered to be the binding region (Fig. 4A). C/EBPβ strongly interacted with MC5R by ChIP measurement *in vitro* (Fig. 4B). C/EBPβ reduced FFA release in α-MSH treatment group compared with that in α-MSH and si-MC5R co-treatment group (Fig. 4C). Figure 4D showed C/EBPβ significantly decreased [^14^C]-palmitate oxidation to CO_2_ in iWAT adipocyte which transfected with si-MC5R and pre-incubated with α-MSH. We found the same data in iBAT adipocytes, which confirmed the negative regulation of C/EBPβ on MC5R in adipocytes fatty acid oxidation (Fig. 4E,F). iWAT and iBAT adipocytes pre-incubated with α-MSH, and co-transfected with pc-C/EBPβ and si-MC5R led to the decrease of CPT-1 and p-ACC compared with those in no pc-C/EBPβ group; CPT-1 activity was also decreased (Fig. 4G,H). Further to explore the negative feedback regulation of C/EBPβ, we pre-incubated adipocytes with α-MSH, and then transfected with pc-C/EBPβ or si-C/EBPβ. Results confirmed negative regulated role of C/EBPβ on MC5R (Figure S4A,C).

### C/EBPβ inhibits adipocytes fatty acid oxidation by negative transcriptional regulation of Foxc2

**Figure 5:**
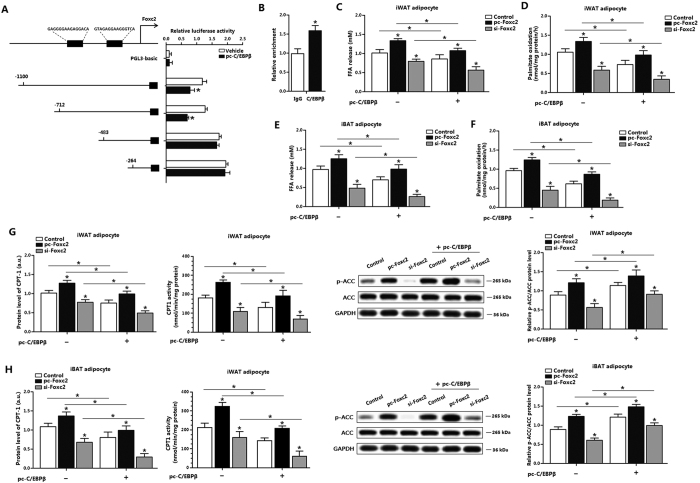
C/EBPβ inhibits adipocytes fatty acid oxidation by negative transcriptional expression of Foxc2. (**A**) Fragments of Foxc2 promoter fused to a luciferase reporter gene were co-transfected into HEK293T cells together with PGL3-basic (control) or pc-C/EBPβ (n = 3). Luciferase activity was corrected for Renilla luciferase activity and normalized to control activity (n = 3). (**B**) Chromatin immunoprecipitation (ChIP) analysis of Foxc2 and C/EBPβ (n = 3). (**C**) Effects of C/EBPβ on FFA level in iWAT adipocytes. Adipocytes were pre-transfected with pc-C/EBPβ, pc-Foxc2 or si-Foxc2 for 72 h (n = 3); (**D**) Effects of C/EBPβ on palmitate oxidation in iWAT adipocytes. Adipocytes were pre-transfected with pc-C/EBPβ, pc-Foxc2 or si-Foxc2 for 72 h (n = 3); (**E**) Effects of C/EBPβ on FFA level in iBAT adipocytes. Adipocytes were pre-transfected with pc-C/EBPβ, pc-Foxc2 or si-Foxc2 for 72 h (n = 3); (**F**) Effects of C/EBPβ on palmitate oxidation in iBAT adipocytes. Adipocytes were pre-transfected with pc-C/EBPβ, pc-Foxc2 or si-Foxc2 for 72 h (n = 3); (**G**) Effects of C/EBPβ on the protein level of CPT-1, CPT-1 activity and phosphorylation level of ACC in iWAT adipocytes. Adipocytes were pre-transfected with pc-C/EBPβ, pc-Foxc2 or si-Foxc2 for 72 h (n = 3); (**H**) Effects of C/EBPβ on the protein level of CPT-1, CPT-1 activity and phosphorylation level of ACC in iBAT adipocytes. Adipocytes were pre-transfected with pc-C/EBPβ, pc-Foxc2 or si-Foxc2 for 72 h (n = 3). pc-C/EBPβ: pcDNA3.1-C/EBPβ overexpression vector of C/EBPβ, pc-Foxc2: adenovirus overexpression vector of Foxc2, si-Foxc2: lentiviral interference vector of Foxc2, CPT-1: carnitine palmitoyl transferase-1, ACC: acetyl-CoA carboxylase, the protein expression of CPT-1 was detected by ELISA test. Values are means ± SD. vs. control group, *p *<* 0.05.

To further examine the relationship between α-MSH and Foxc2, we used the same detection method to explore whether C/EBPβ and Foxc2 interacted directly. With Genomatix software analyzation, we found two potential binding sites of C/EBPβ on the Foxc2 promoter region (Fig. 5A). And with luciferase reporter assay, the −712~−483 region was considered as the binding region (Fig. 5A). ChIP analysis showed C/EBPβ strongly interacted with Foxc2 (Fig. 5B). FFA level in pc-Foxc2 and pc-CEBP/β co-transfected group decreased to normal level compared with that in pc-Foxc2 group (Fig. 5C). Measurement of [^14^C]-palmitate oxidation to CO_2_ indicated CEBP/β inversed the effect of Foxc2, suggesting C/EBPβ played a negative regulation role on Foxc2; and CEBP/β reduced fatty acid oxidation (Fig. 5D). Similar observations were obtained in iBAT adipocytes, which confirmed the negatively regulation of CEBP/β on Foxc2 (Fig. 5E,F). Compared with that in pc-Foxc2 treatment group, CPT-1 was decreased in pc-C/EBPβ and pc-Foxc2 co-transfected group, but the phosphorylation of ACC was decreased; and CPT-1 activity was also decreased (Fig. 5G,H). Further to explore the negative feedback regulation of C/EBPβ, we pre-incubated adipocytes with pc-Foxc2, and then transfected with pc-C/EBPβ or si-C/EBPβ. Results indicate C/EBPβ negative regulates of Foxc2 (Figure S4B,D).

### α-MSH and Foxc2 promote fatty acid oxidation via cAMP/PKA signal pathway in adipocytes

**Figure 6:**
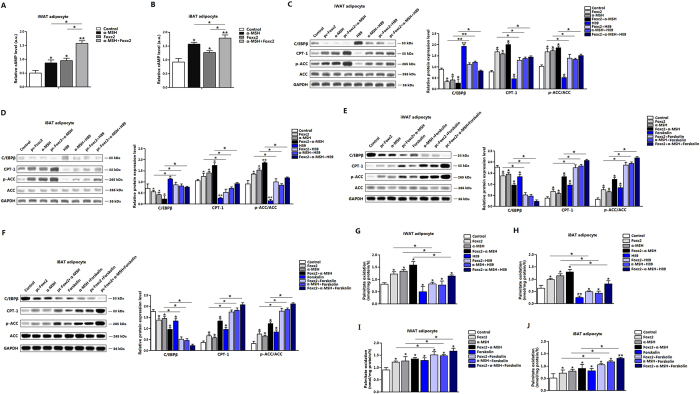
α-MSH and Foxc2 promote fatty acid oxidation via cAMP/PKA signal pathway in adipocytes. (**A**) cAMP level of iWAT adipocytes. Cells were pre-transfected with pc-Foxc2 for 72 h, and then incubated with 500 nM α-MSH for 1 h (n = 3); (**B**) cAMP level of iBAT adipocytes. Cells were pre-transfected with pc-Foxc2 for 72 h, and then incubated with 500 nM α-MSH for 1 h (n = 3); (**C**) Protein levels of C/EBPβ, CPT-1 and p-ACC of iWAT adipocytes. Cells were pre-transfected with pc-Foxc2 for 72 h, and then incubated with α-MSH or H89 (n = 3); (**D**) Protein levels of C/EBPβ, CPT-1 and p-ACC of iBAT adipocytes. Cells were pre-transfected with pc-Foxc2 for 72 h, and then incubated with α-MSH or H89 (n = 3); (**E**) Protein levels of C/EBPβ, CPT-1 and p-ACC of iWAT adipocytes. Cells were pre-transfected with pc-Foxc2 for 72 h, and then incubated with α-MSH or Forskolin (n = 3); (**F**) Protein levels of C/EBPβ, CPT-1 and p-ACC of iBAT adipocytes. Cells were pre-transfected with pc-Foxc2 for 72 h, and then incubated with α-MSH or Forskolin (n = 3); (**G**) Palmitate oxidation in iWAT adipocytes. Cells were pre-transfected with pc-Foxc2 for 72 h, and then incubated with α-MSH or H89 (n = 3); (**H**) Palmitate oxidation in iBAT adipocytes. Cells were pre-transfected with pc-Foxc2 for 72 h, and then incubated with α-MSH or H89 (n = 3); (**I**) Palmitate oxidation in iWAT adipocytes. Cells were pre-transfected with pc-Foxc2 for 72 h, and then incubated with α-MSH or Forskolin (n = 3); (**J**) Palmitate oxidation in iBAT adipocytes. Cells were pre-transfected with pc-Foxc2 for 72 h, and then incubated with α-MSH or Forskolin (n = 3). pc-Foxc2: adenovirus overexpression vector of Foxc2, CPT-1: carnitine palmitoyl transferase-1, ACC: acetyl-CoA carboxylase, Values are means ± SD. vs. control group, *p < 0.05, **p *<* 0.01.

MC5R is regarded as a G-protein-coupled receptor, and it linked to cAMP generation via stimulating G-proteins^28^. To delineate molecular mechanism underlies the regulation of α-MSH and Foxc2 on adipocyte fatty acid oxidation, we measured the level of cAMP. Figure 6A showed α-MSH and Foxc2 both elevated cAMP, and co-treatment of α-MSH and Foxc2 presented a higher level of cAMP (Fig. 6A). Same observations were showed in iBAT adipocytes while the effect of Foxc2 was not strong compared with that in α-MSH treatment group (Fig. 6B). Figure 6C showed addition of H89 blocked CPT-1 and p-ACC, but elevated CEBP/β. pc-Foxc2 and α-MSH together had the opposite effects. Adipocytes treated with the combination of pc-Foxc2, α-MSH and H89 increased CPT-1 and p-ACC (Fig. 6C). In iBAT adipocytes H89 decreased the CPT-1 and p-ACC and the addition of pc-Foxc2 and α-MSH reversed these results effectively (Fig. 6D). In forsklin treatment group, CEBP/β was reduced, but CPT-1 and p-ACC were increased. And in the combination treatment of pc-Foxc2, α-MSH and Forsklin group, CPT-1 and p-ACC were increased the most compared with those in control group (Fig. 6E). Same data were shown in iBAT adipocytes by Forsklin (Fig. 6F). H89 decreased [^14^C]-palmitate oxidation to CO_2_, while after pc-Foxc2 and α-MSH treatment the production of CO_2_ increased to a statistical significance level (Fig. 6G,H). Forskolin increased the production of CO_2,_ and co-treatment of forskolin and pc-Foxc2 further increased this effect (Fig. 6I,J). In addition, co-treatment of pc-Foxc2 and α-MSH synergistically promoted forskolin-induced palmitate oxidation. Thus, our data demonstrate Foxc2 and α-MSH combined together in promoting fatty acid oxidation via cAMP/PKA pathway in both white and brown adipocytes.

## Discussion

The melanocortin pathway is important in the regulation of energy homeostasis, food intake and body weight^21^,^29^. Melanocortins appear to regulate energy homeostasis by two distinct pathways: sympathetic nervous system innervation or directly by circulating melanocortins^30^. In the CNS, α-MSH can directly regulate lipid metabolism by binding to its receptor in the hypothalamus. Recent studies show that melanocortins pathway in the CNS controls nonexercise activity thermogenesis and hepatic glucose production^31^,^32^. Moreover, reduced α-MSH level in CNS promotes adiposity and impairs lipolysis^33^.

α-MSH also control energy expenditure and lipid metabolism in peripheral tissues and organs, especially in adipose tissue by binding to specific receptors^34^,^35^,^36^. Recent studies show MC5R regulates adipocytes differentiation in MC5R-null mice, which consistent with previously findings in rodents^20^,^37^. In fact, α-MSH can also directly stimulate adipose tissue lipolysis via MC5R and cAMP/PKA signal pathway^20^,^38^. So we assume that the α-MSH/MC5R-mediated lipolysis may be associated with fatty acid oxidation in adipocytes. Here, we found intraperitoneal injection of α-MSH significantly reduced [^14^C]-palmitate incorporation into different adipose tissues (iWAT, eWAT, iBAT). α-MSH also elevated the activity of CPT-1, and increased fatty acid oxidation most effectively during lipolysis in white and brown adipose tissues. Meanwhile, α-MSH elevated the mRNA levels of MC5R and Foxc2. These data suggested α-MSH exhibits positive regulation on fatty acid oxidation in adipose tissue and Foxc2 effects during this process.

Foxc2 is originally found in mice embryo trunk, head, and limbs in the mesoderm^39^. Foxc2 regulates fat metabolism and improves insulin sensitivity via various obese signaling pathways^26^,^40^,^41^. In our present study we showed Foxc2 decreased [^14^C]-palmitate incorporation into white and brown adipose tissues. CPT-1 activity and phosphorylation of ACC were increased in iWAT and iBAT. This effect was more obvious in iBAT which has more energy expenditure and thermogenesis. Our results indicated α-MSH and Foxc2 had the same effects on fatty acid oxidation in white and brown adipose tissues.

Mitochondrial β-oxidation is one of the major biochemical factors to maintain adipocytes function in both white and brown adipocytes^42^,^43^. Increased FAO leads to a marked increase of nuclear genes encoding mitochondrial fatty acid β-oxidation enzymes and causes a dramatic increase in CO_2_ production. By treating white and brown adipocytes with α-MSH and Foxc2, we observed that these two factors combined together to promote FFA release and increase palmitate oxidation to CO_2_. The mitochondrial UCP, a distinctive and specific marker for FAO, has been a major factor on the transcriptional control of MCAD, LCAD and other-oxidation enzymes *in vivo*^44^,^45^. Our data showed α-MSH and Foxc2 elevated MCAD and LCAD levels, along with the elevation of CPT-1, p-ACC, and PGC1-α. Additionally, C/EBPβ was significantly inhibited by α-MSH and Foxc2 alone- or co-treatment in white and brown adipocytes.

C/EBPβ plays an important role in pre-adipocyte differentiation and adipogenesis^46^,^47^. However, there is little known about its function on fatty acid oxidation. In this study, we found C/EBPβ acted as a transcription regulator of Foxc2 and MC5R, and bind with the promoter region of these two genes. Moreover, the interaction of C/EBPβ with Foxc2 and MC5R reduced CPT-1 and blocked fatty acid oxidation. MCRs belong to the G-protein-couple receptor family; they are all linked to cAMP generation via stimulating G-proteins and adenylate cyclase^19^,^48^. And their down-stream PKA activation could stimulate the activity of CPT-1^49^,^50^. Our results showed α-MSH and Foxc2 increased cAMP level and activated PKA signal. While H89, a specific inhibitor of PKA signal, significantly blocked this effect and inhibited fatty acid oxidation.

In summary, our study provided new insights into the mechanisms required for the regulation of α-MSH and Foxc2 on fatty acid oxidation in white and brown adipose tissues. Our data indicated α-MSH and Foxc2 were mediated by cAMP/PKA activation, and C/EBPβ was a novel transcriptional suppressor of MC5R and Foxc2 (Figure S5). Our findings investigate that α-MSH affects the adipocyte FAO, make fundamental for the direct regulation of CNS on peripheral FAO and these findings will promise as a novel strategy for treatment of obesity-associated metabolic complications.

## Methods

### Animal experiment

Three-week old C57BL/6J male mice were purchased from the Laboratory Animal Center of the Fourth Military Medical University (Xi’an, China). All mice handling methods and all experimental protocols were conducted following guidelines and regulations approved by the Animal Ethics Committee of Northwest A&F University. Mice were provided *ad libitum* water and standard laboratory chow diet purchased from Animal Center of the Fourth Military Medical University. Animal room was maintained at 25 ± 1 °C, humidity at 55 ± 5%, and a 12 h light/12-dark cycle. Four-week old mice were injected either PBS or α-MSH (500 nM) intraperitoneally once a day for two days before dark cycle. Inguinal adipose tissue (iWAT) was removed at 0.5 h, 1 h, 2 h, 4 h and 6 h after the last injection from the treatment group. Vehicle, si-Foxc2 and pc-Foxc2 were administered as a daily intraperitoneal injection for 6 days. Tissue samples of epididymal white adipose tissue (eWAT), inguinal WAT (iWAT), and interscapular brown fat (iBAT) pads were removed for future studies.

### Primary adipocyte culture

For collected iWAT samples, the connective fiber and blood vessels were removed and washed three times with PBS buffer containing 200 U/mL penicillin (Sigma, St. Louis, USA) and 200 U/mL streptomycin (Sigma, St. Louis, USA). The adipocyte culture was carried out according to our previous publication. Briefly, pre-adipocytes were seeded onto 35-mm culture dishes at a density of 8 × 10^4^ cells/dish, and incubated at 37 °C under a humidified atmosphere of 5% CO_2_ and 95% air until confluence. Differentiation of white pre-adipocytes was performed as following. Cells grown to 100% confluence (Day 0) were exposed to the induction DMEM/F12 containing dexamethasone (1 μM; Sigma), insulin (10 μg/mL; Sigma), IBMX (0.5 mM, Sigma) and 10% FBS. Four days after the induction (from Day 2), cells were maintained in the induction medium containing insulin (10 μg/mL, Sigma) and 10% FBS until the day for cell harvest. And brown adipocytes were induced to differentiate using DEME/F12 with 15% FBS, 0.5 mM IBMX; 0.25 mM indomethacin; 2 μg/mL dexamethasone; 1 nM T3, 20 nM insulin for 2 days and subsequently maintained on differentiation media (1 nM T3, 20 nM insulin).

### Materials and vectors

α-MSH was purchased from Sigma (St. Louis, MO, USA), and the working solution (500 nM) was prepared to treat adipocytes for 1 h. The recombinant adenovirus overexpression vector of Foxc2 (pc-Foxc2), recombinant lentiviral interference vector of Foxc2 (si-Foxc2), and lentiviral interference vector of MC5R (si-MC5R) were constructed in our lab. The overexpression plasmid vector of C/EBPβ (pc-C/EBPβ) and interference vector of C/EBPβ (si-C/EBPβ) were constructed by the Shanghai GenePharma Co., Ltd (Shanghai, China). The adenovirus and lentiviral recombinant vectors were infection in a titer of 1 × 10^9^ IFU/mL for Foxc2. The *in vivo* experiments were performed after 6–8 days of infection. H89 and Forsklin were bought from Selleck.cn (Selleck Chemicals, USA). Thirty minutes before α-MSH stimuli, H89 (10 μM) or Forskolin (10 μM) were used to treat adipocytes. cAMP was measured using the FlashPlate^TM^ method according to the FlashPlate (Perkin Elmer, USA) protocol.

### Fatty acid oxidation measurement

Palmitate oxidation to CO_2_ by tissue homogenate and the incorporation of palmitate into lipids were measured according to previous published method^51^,^52^,^53^,^54^. In brief, adipose tissues was homogenized in sucrose/Tris/EDTA buffer, and incubated for 60 min in the reaction mixture which contained [1-^14^C] palmitic acid and measured for trapped CO_2_. For *in vivo* study of palmitate oxidation to CO_2_, we used the modified method from Sebastián and our pervious study^55^,^56^. For the trapped radioactive CO_2_ measurement, a parafilm-sealed system was used. The reaction was stopped by the addition of 40% perchloric acid through a syringe that pierced the parafilm. Serum and adipocytes triglycerides (TG) and free fatty acids (FFA) contents were determined by using commercial ELISA kits (Jiancheng, China) according to the instructions.

### Measurement of CPT-1 activity

The CPT-1 activity assay was performed as described by Pawel and Bremer^57^. In brief, mitochondria of adipose tissue or adipocytes were first isolated using the commercial isolation kits form Abcam (ab110170 and ab110168; Cambridge, UK) according to the protocols. Then 200 μg of mitochondrial protein was added to the assay medium (20 mM HEPES, 75 mM KCl, 2 mM KCN, 1% fat free BSA, 70 μM palmitoyl-CoA, 0.25 mM L-[^3^H]carnitine) with or without different levels of malonyl-CoA. Samples were incubated at 37 °C for 3 min. And then stopped by the addition of 0.5 ml of ice-cold perchloric acid. After mitochondrial centrifuged, the mitochondrial pellet was then resuspended in ddH_2_O and extracted with 600 μL of butanol 300 μL of the butanol-phase was counted by liquid scintillation.

### Enzyme-linked immunosorbent (ELISA) assay

Serum α-MSH, CPT-1, MCAD and LCAD were measured using commercial ELISA kits (R&D Systems, USA). Adipose tissues were collected as described previously. Cells were collected and disrupted by ultrasonication (28 KHz, 30 min).

### Chromatin Immunoprecipitation (ChIP) assays

Adipocytes were prepared for chromatin immunoprecipitation (ChIP) assay using a ChIP assay kit (Abcam, Cambridge, UK) according to the manufacturer’s protocol. Crosslinking was reversed, and purified DNA was subjected to Real-time PCR with SYBR green fluorescent dye (Invitrogen, USA).

### Promoter reporter assay and dual luciferase reporter assay

Four fragments containing Foxc2–5′ sequences from −1100 to −264 relative to the transcription initiation site were sub-cloned into pGL3-basic vector (Takara, China). HEK293T cells were cultured in 24-well plates and co-transfected with Foxc2 promoter plasmids and pGL3-basic plasmid (control reporter). Cells were harvested 48 h after transfection, and detected using the Dual-Luciferase Reporter assay system (Promega, USA). The MC5R promoter reporter assay and luciferase reporter assay were performed as the same protocol. And the four fragments containing MC5R-5′ sequences were from −1200 to −210 relative to the transcription initiation site.

### Real-time quantitative PCR analysis

Total RNA from adipose tissues and cells were extracted with TRIpure Reagent kit (Takara, China). 400 ng of total RNA was reverse transcribed using M-MLV reverse transcriptase kit (Takara, China). Primers were synthesized by Shanghai Sangon Ltd (Shanghai, China). Quantitative PCR was performed in 25 μL reaction system containing specific primers and SYBR Premix EX Taq (Takara, Dalian, China). The levels of mRNA were normalized using β-actin. The expression of genes was analyzed by method of 2^−ΔΔCt^.

### Immunoblot analysis

Mice pre-adipocytes and adipose tissues were solubilized in lysing buffer. The solubilization was preceded for 40 min at 4 °C, then the solution was centrifuged at 12,000× g for 15 min at 4 °C, and the supernatants were used for determination of protein concentration. Protein samples (30 μg) were separated by electrophoresis on 12% and 5% SDS-PAGE gels using slab gel apparatus, and transferred to PVDF nitrocellulose membranes (Millipore, USA), blocked with 5% skim milk powder/Tween 20/TBST at room temperature for 2 h. The membranes were then incubated with primary antibodies against CPT-1, p-ACC, ACC, Cidea, PGC1-α, FABP3, FABP4, UCP1, UCP2 and GAPDH (Bioworld, China) at 4 °C overnight. Following this step, the appropriate HRP-conjugated secondary antibodies (Boaoshen, China) were added and incubated for 2 h at room temperature. Proteins were visualized using chemiluminescent peroxidase substrate (Millipore, USA), and then the blots were quantified using ChemiDoc XRS system (Bio-Rad, USA).

### Statistical analysis

Statistical analyses were conducted using SAS v8.0 (SAS Institute, Cary, NC). Data were analyzed using either one-way ANOVA or two-way ANOVA. Comparisons among individual means were made by Fisher’s least significant difference (LSD). Data were presented as mean ± SD. *p* *<* 0.05 was considered to be significant.

## Additional Information

**How to cite this article**: Gan, L. *et al*. α-MSH and Foxc2 promote fatty acid oxidation through C/EBPβ negative transcription in mice adipose tissue. *Sci. Rep.* **6**, 36661; doi: 10.1038/srep36661 (2016).

**Publisher’s note:** Springer Nature remains neutral with regard to jurisdictional claims in published maps and institutional affiliations.

## Supplementary Material

Supplementary Information
