# Supplementary material for: α-MSH and Foxc2 promote fatty acid oxidation through C/EBPβ negative transcription in mice adipose tissue
Source: Sci Rep. 2016 Nov 7;6:36661. doi: 10.1038/srep36661 (PMC5098202; doi:10.1038/srep36661)
Supplement: Supplementary Information [file srep36661-s1.doc]

**Supplement Information**

**α-MSH** **and Foxc2 promote fatty acid oxidation through C/EBPβ negative transcription in mice adipose tissue**

Lu Gan, Zhenjiang Liu, Yizhe Chen, Dan Luo, Fei Feng, Guannv Liu, Chao Sun

**Supplement Experiment**


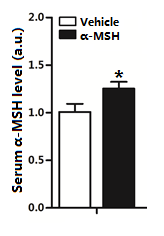
**Figure S1:**

**Fig.S1 Relative serum** **α-MSH level (Related to Fig.1).** Serum α-MSH level was measured after 500 nM α-MSH injection for 1 h (n=10). Values are means ± SD. vs. control group, * *p* < 0.05.


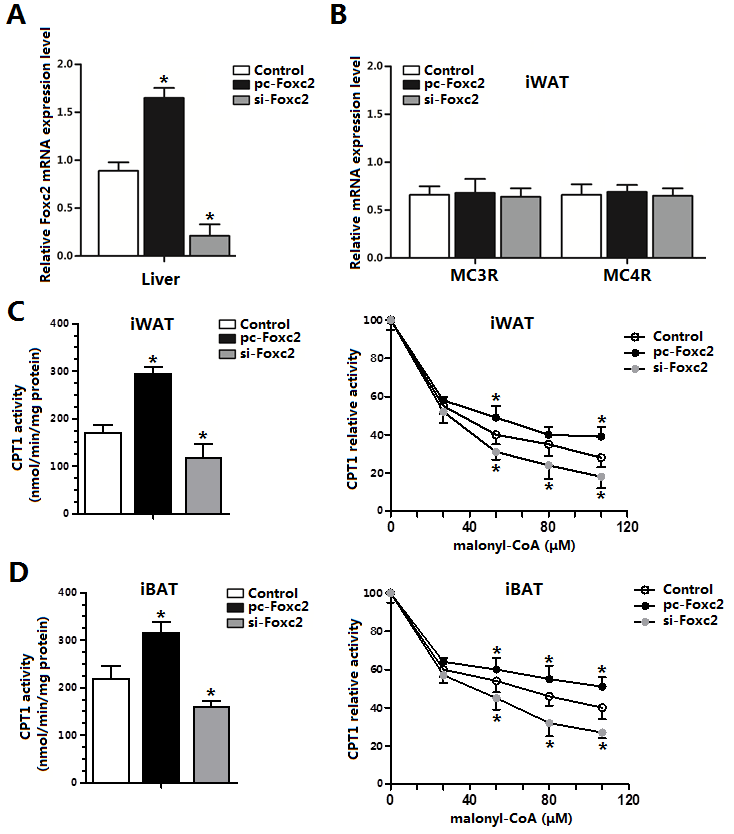
**Figure S2:**

**Fig.S2** (A) Relative mRNA expression of Foxc2 in liver (Related to Fig. 2-3, 5-6)**.** mRNA expression of Foxc2 in liver was measured after pc-Foxc2 or si-Foxc2 intraperitoneal injection for 6 days (n=6). (B) mRNA levels of MC3R and MC4R of iWAT was measured after pc-Foxc2 or si-Foxc2 intraperitoneal injection for 6 days (n=6). (C) Left: CPT-1 activity of iWAT after pc-Foxc2 or si-Foxc2 intraperitoneal injection for 6 days (n=6); right: CPT-1 sensitivity to malonyl-CoA inhibition of iWAT after pc-Foxc2 or si-Foxc2 intraperitoneal injection for 6 days (n=6). (D) Left: CPT-1 activity of iBAT after pc-Foxc2 or si-Foxc2 intraperitoneal injection for 6 days (n=6); right: CPT-1 sensitivity to malonyl-CoA inhibition of iBAT after pc-Foxc2 or si-Foxc2 intraperitoneal injection for 6 days (n=6).Control group: no transfection group, pc-Foxc2: recombinant adenovirus overexpression vector of Foxc2, si-Foxc2: recombinant lentiviral interference vector of Foxc2.Values are means ± SD. vs. control group, * *p* < 0.05.


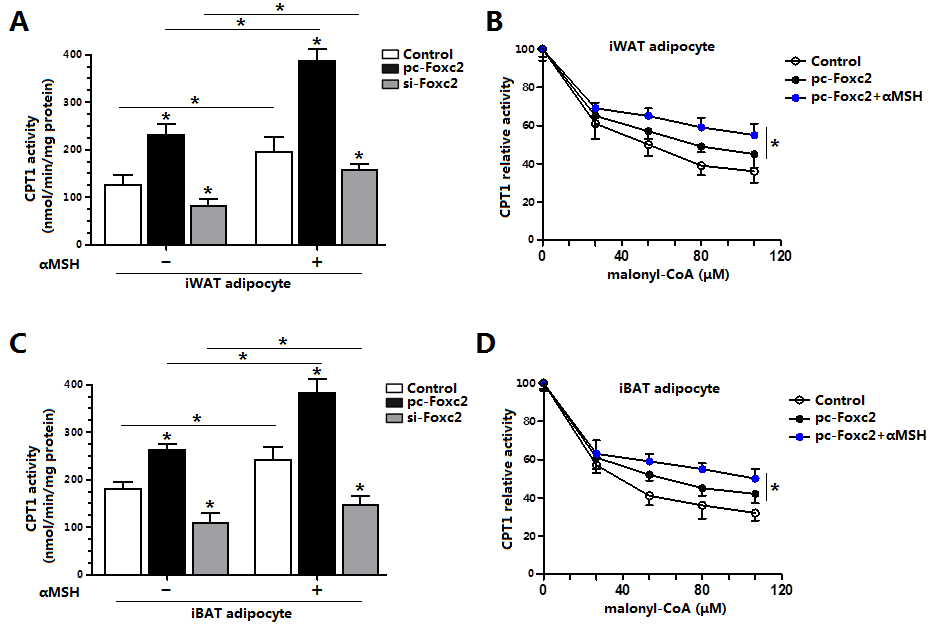
**Figure S3:**

**Fig.S3 CPT-1 activity measurement (Related to Fig. 3).** (A) CPT-1 activity of iWAT adipocytes pre-treated with pc-Foxc2 or si-Foxc2, and then incubated with α-MSH or not (n=3). (B) CPT-1 sensitivity to malonyl-CoA inhibition of iWAT adipocytes pre-treated with pc-Foxc2 or si-Foxc2, and then incubated with α-MSH or not (n=3). (C) CPT-1 activity of iBAT adipocytes pre-treated with pc-Foxc2 or si-Foxc2, and then incubated with α-MSH or not (n=3). (D) CPT-1 sensitivity to malonyl-CoA inhibition of iWAT adipocytes pre-treated with pc-Foxc2 or si-Foxc2, and then incubated with α-MSH or not (n=3). Control group: no transfection group, pc-Foxc2: recombinant adenovirus overexpression vector of Foxc2, si-Foxc2: recombinant lentiviral interference vector of Foxc2.Values are means ± SD. vs. control group, * *p* < 0.05.


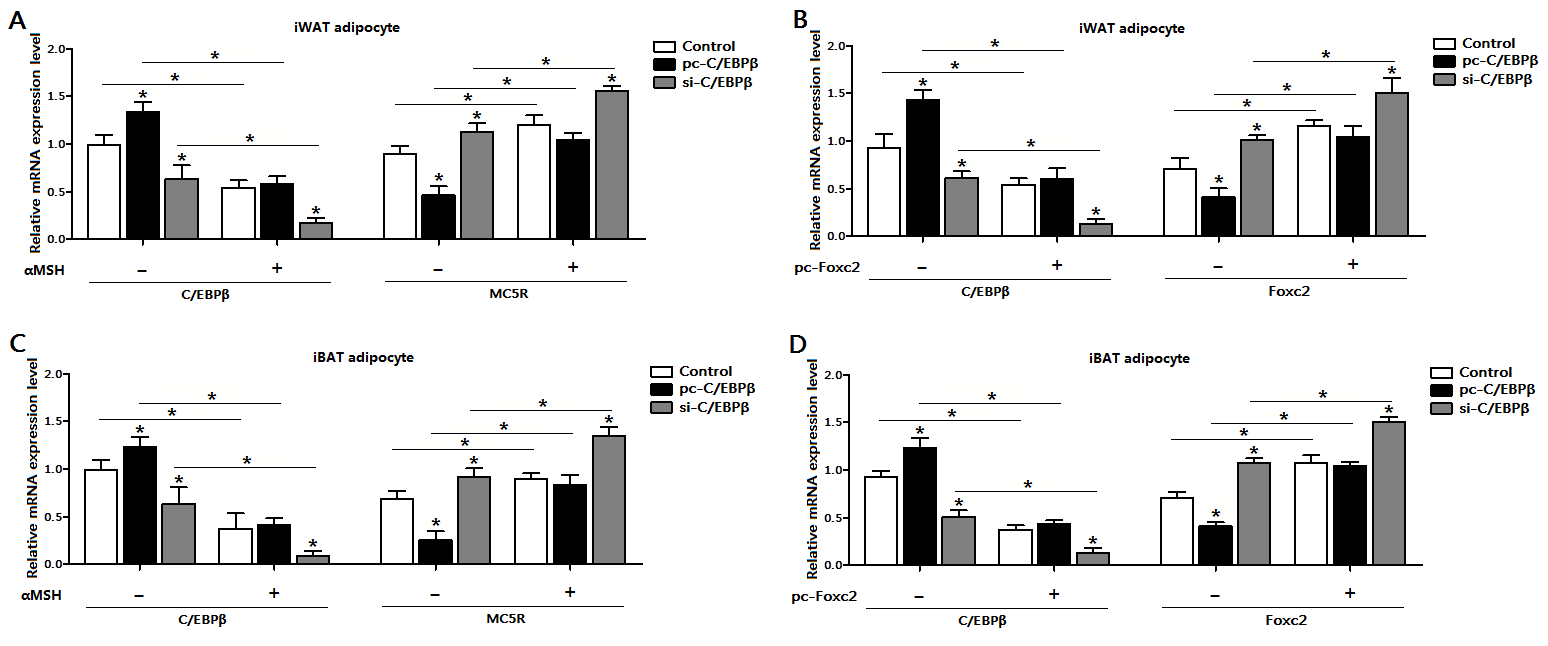
**Figure S4:**

**Fig.S4 Relative mRNA expression of C/EBPβ, MC5R and Foxc2 (Related to Fig.4-5).** (A) Relative mRNA expression levels of C/EBPβ and MC5R incubation with or without 500 nM α-MSH for 1h. Adipocytes from iWAT were pre-transfected with pc-C/EBPβ or si-C/EBPβ for 72h (n=3). (B) Relative mRNA expression levels of C/EBPβ and Foxc2. Adipocytes from iWAT were transfected with pc-C/EBPβ or si-C/EBPβ or pc-Foxc2 for 72 h (n=3). (C) Relative mRNA expression levels of C/EBPβ and MC5R incubation with or without 500 nM α-MSH for 1h. Adipocytes from iBAT were pre-transfected with pc-C/EBPβ and si-C/EBPβ for 72 h (n=3). (D) Relative mRNA expression levels of C/EBPβ and Foxc2. Adipocytes from iBAT were transfected with pc-C/EBPβ or si-C/EBPβ or pc-Foxc2 for 72 h (n=3). Control group: no transfection group, pc-Foxc2: recombinant adenovirus overexpression vector of Foxc2, pc-C/EBPβ: the overexpression plasmid vector of C/EBPβ, si-C/EBPβ: interference vector of C/EBPβ. Values are means ± SD. vs. control group, * *p* < 0.05.

**Figure S5:**


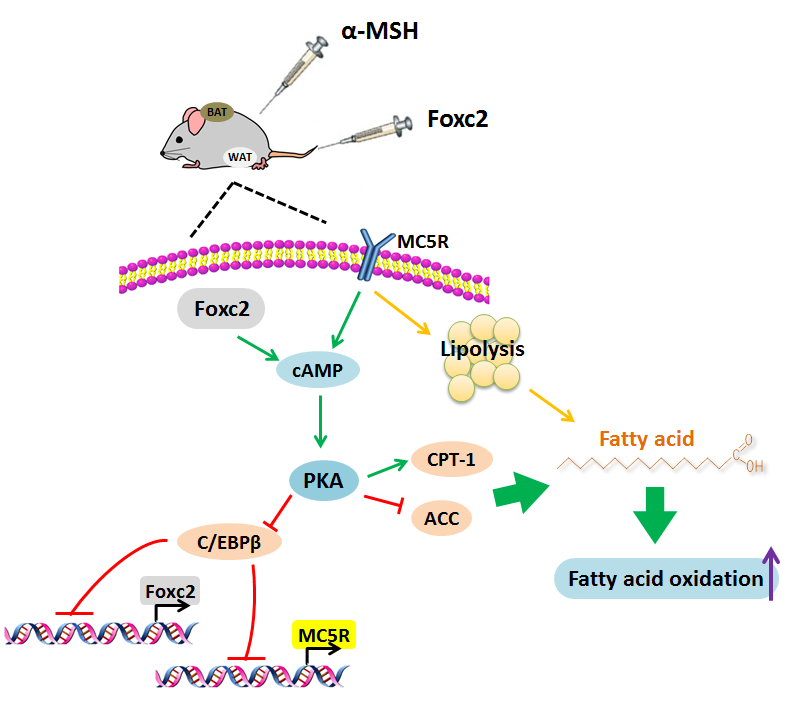
**Fig.S5** Summary of α-MSH and Foxc2 jointly promote fatty acid oxidation in white and brown adipose via cAMP/PKA signal pathway and negatively transcription regulation of C/EBPβ.


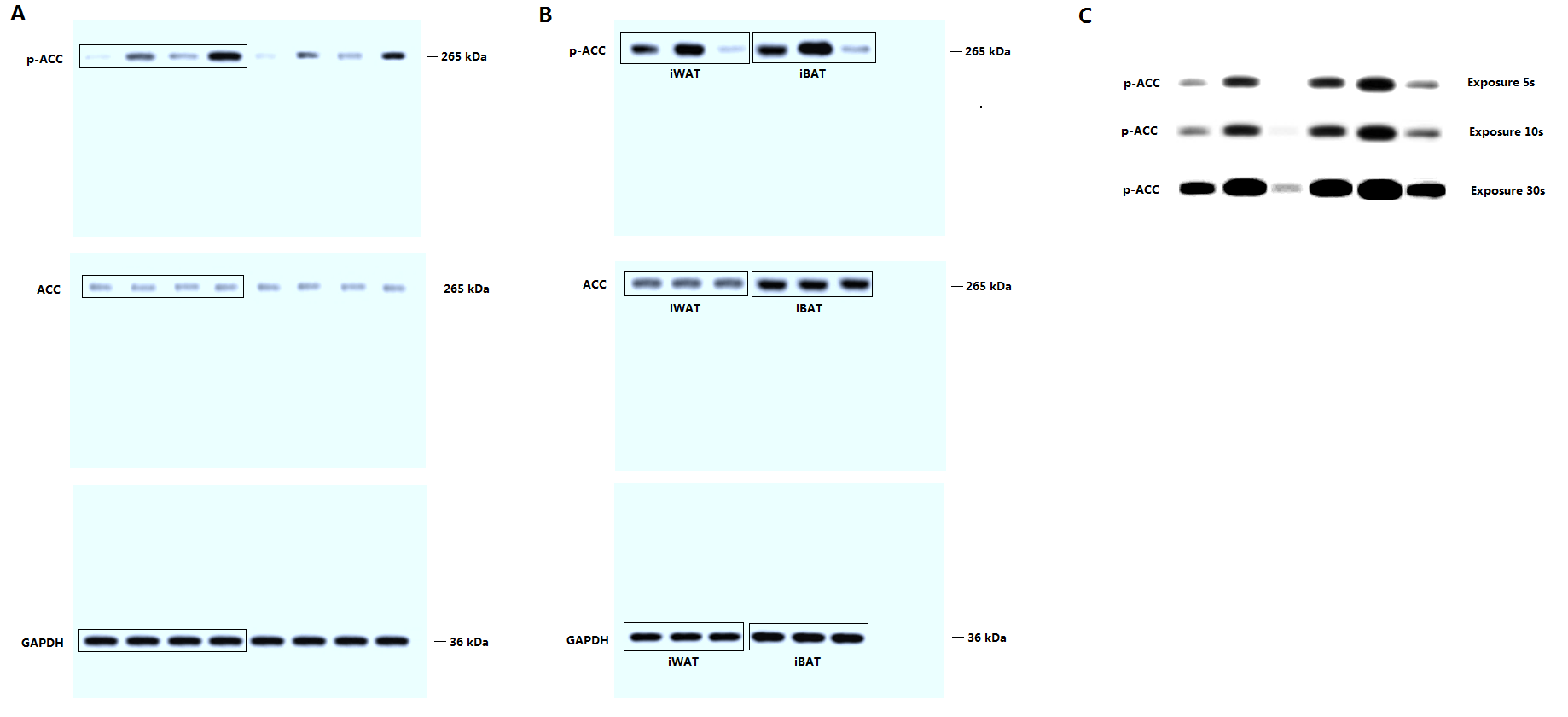
**Figure S6:**

**Fig. S6** (Related with Fig 1, 2 and 5) (A) The full-length blots of ACC and p-ACC in Fig.1H. (B) The full-length blots of ACC and p-ACC in Fig. 2G and 2I. (C) Multiple exposures of p-ACC in Fig. 5H.
